# Perturbing glycosylphosphatidylinositol (GPI)-anchor biosynthesis alters cell wall architecture and modulates fungal morphology

**DOI:** 10.64898/2026.06.05.730525

**Published:** 2026-06-09

**Authors:** Hui Ting Chu, Isha Gautam, Surya Pavan Yenamendra, Tuo Wang, Prakash Arumugam

## Abstract

Filamentous fungi are widely used as expression hosts for industrial protein production. However, their tendency to form dense mycelial pellets limits nutrient transfer, oxygen uptake, and fermentation efficiency. While cell wall components are known to influence fungal aggregation, the molecular mechanisms linking cell wall biosynthesis to macroscopic morphology remain poorly defined. Here, we show that perturbation of glycosylphosphatidylinositol (GPI)-anchored protein biosynthesis influences fungal morphology by altering cell wall composition. Using *Aspergillus oryzae* as a model system, we demonstrate that disruption of the GPI ethanolamine phosphate transferase 1 ( *mcd4)* or inhibition of glucosaminyl phosphatidylinositol acyltransferase Gwt1 using antifungal drug manogepix (MGX) induces a hyper-branching phenotype and weakens cell wall. Notably, chemical inhibition of Gwt1 by MGX caused a marked transition from pelleted to dispersed mycelial growth. Solid-state NMR (ssNMR) analysis revealed reorganisation of galactosaminogalactan (GAG) and galactomannan (GM), including complete loss of cationic galactosamine (GalN), a key determinant of hyphal adhesion. Transcriptomic profiling further revealed downregulation of genes involved in somatic cell fusion, linking altered wall composition to impaired germling aggregation. Strikingly, MGX treatment induced distinct morphological outcomes in other industrially relevant fungi, indicating species-specific cell-wall dependencies. Together, our findings establish GPI-anchored cell wall proteins as important regulators of fungal morphology and provide new strategies for rational morphology engineering in industrial fermentation.

## Full Text Availability

The license terms selected by the author(s) for this preprint version do not permit archiving in PMC. The full text is available from the preprint server.

