# Supplementary material for: Perturbing glycosylphosphatidylinositol (GPI)-anchor biosynthesis alters cell wall architecture and modulates fungal morphology
Source: bioRxiv. 2026 Jul 5:2026.06.05.730525. Originally published 2026 Jun 9. Preprint. [Version 2] doi: 10.64898/2026.06.05.730525 (PMC13278039; doi:10.64898/2026.06.05.730525)
Supplement: Supplement 1 [file media-1.pdf]

Supplementary Information

Perturbing glycosylphosphatidylinositol (GPI)-anchor biosynthesis alters cell wall architecture and modulates fungal morphology

Hui Ting Chu[a, b], Isha Gautam[c], Surya Pavan Yenamandra[d], Tuo Wang[c], Prakash Arumugam[a,e, *]

[a] Singapore Institute of Food and Biotechnology Innovation (SIFBI), Agency for Science, Technology and Research (A*STAR), 31 Biopolis Way, Nanos, Singapore 138669, Singapore.

[b] Department of Chemistry, National University of Singapore, 3 Science Drive 3, Singapore 117543, Singapore

[c] Department of Chemistry, Michigan State University, East Lansing, MI 48824, USA

[d] Environmental Health Institute, 11 Biopolis Way, Singapore 138667, Singapore.

[e] Nanyang Technological University, School of Biological Sciences, Singapore 637551, Singapore

*Corresponding Author

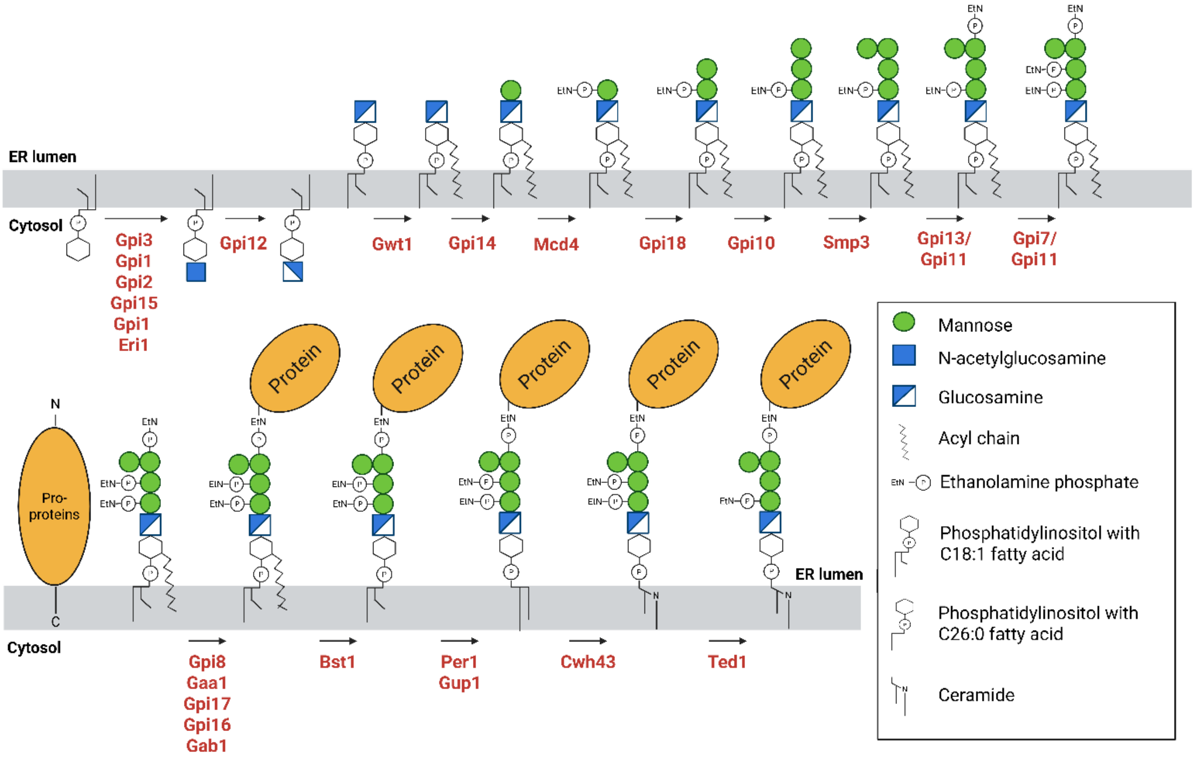

Supplementary Fig. 1: Glycosylphosphatidylinositols (GPI)-anchor protein biosynthesis at the endoplasmic reticulum (ER) of yeast and filamentous fungi. Created with BioRender.com.

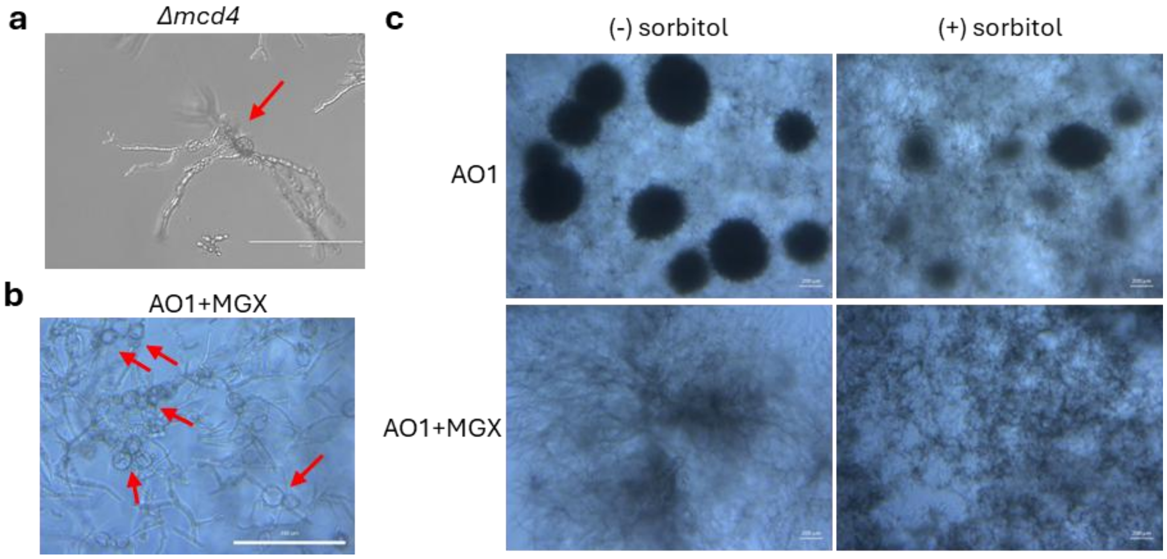

Supplementary Fig. 2: Perturbation of GPI-anchor biosynthesis pathway alters morphology of *A. oryzae* AO1. (a) The Δ*mcd4* hyphae exhibit swelling, hyperbranching, and cell wall rupture with release of cytosolic contents (red arrow). Scale bar, 200 µm. (b) MGX treatment induces swollen hyphae in AO1. Scale bar, 1000 µm. (c) Addition of 1.2 M sorbitol to AO1 + 0.5 mg/L MGX did not rescue the pellet morphology after 4 days of growth. Scale bar, 200 µm.

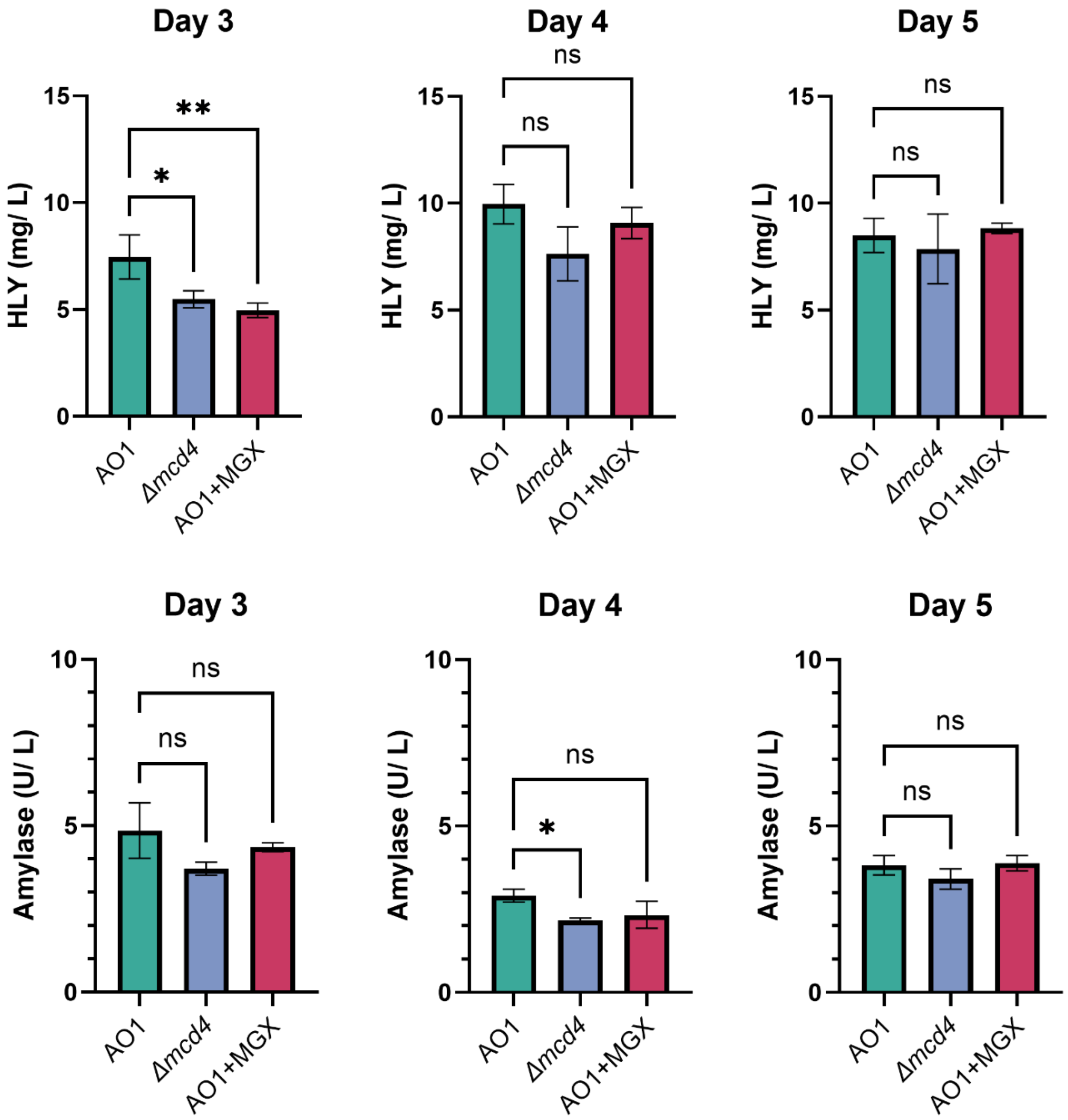

Supplementary Fig. 3: Effects of chemical and genetic perturbation of GPI-AP on recombinant (HLY) and native (α-amylase) protein secretion.

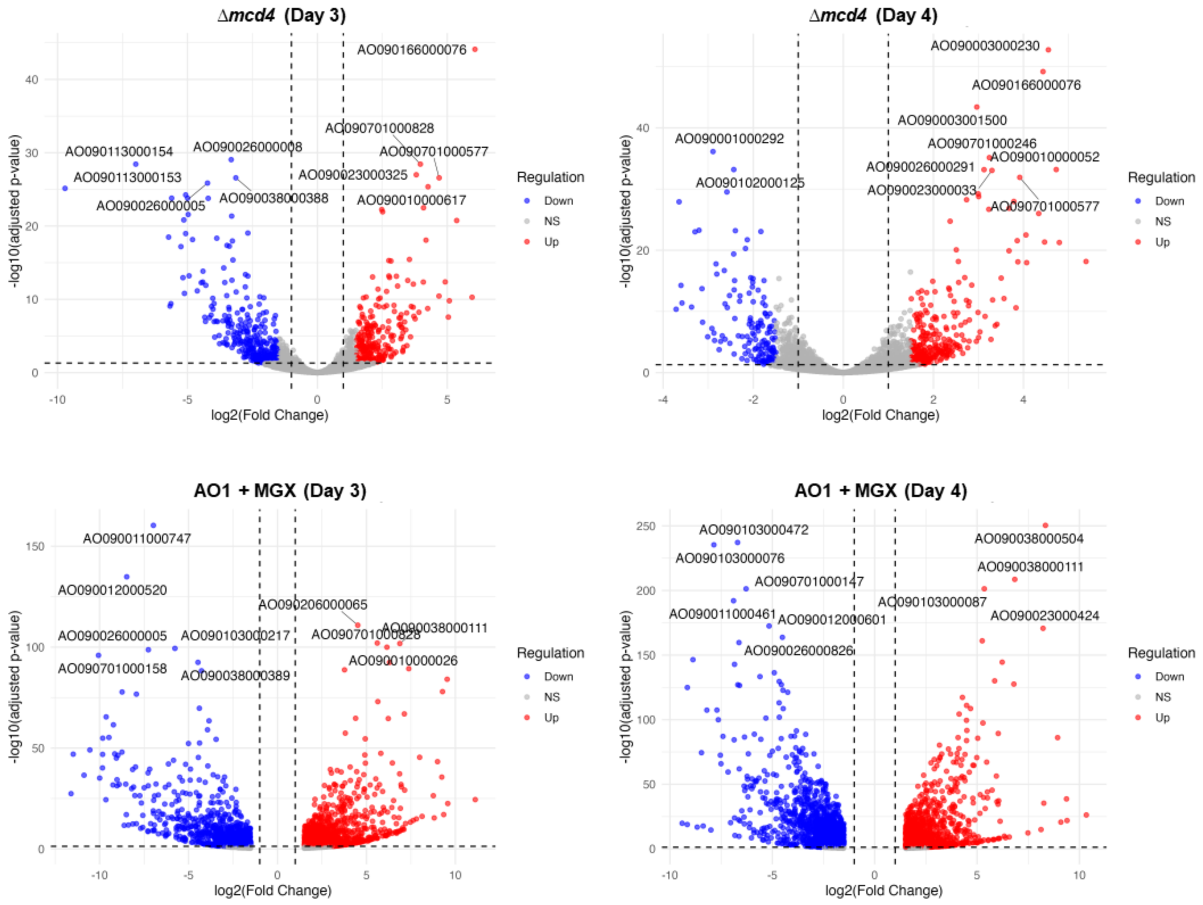

Supplementary Fig. 4: Volcano plot of DEGs identified in Δ*mcd4* and MGX-treated AO1 relative to AO1 on days 3 and 4, showing that DEGs in MGX-treated AO1 have a wider range of $\log_2$ fold change values.

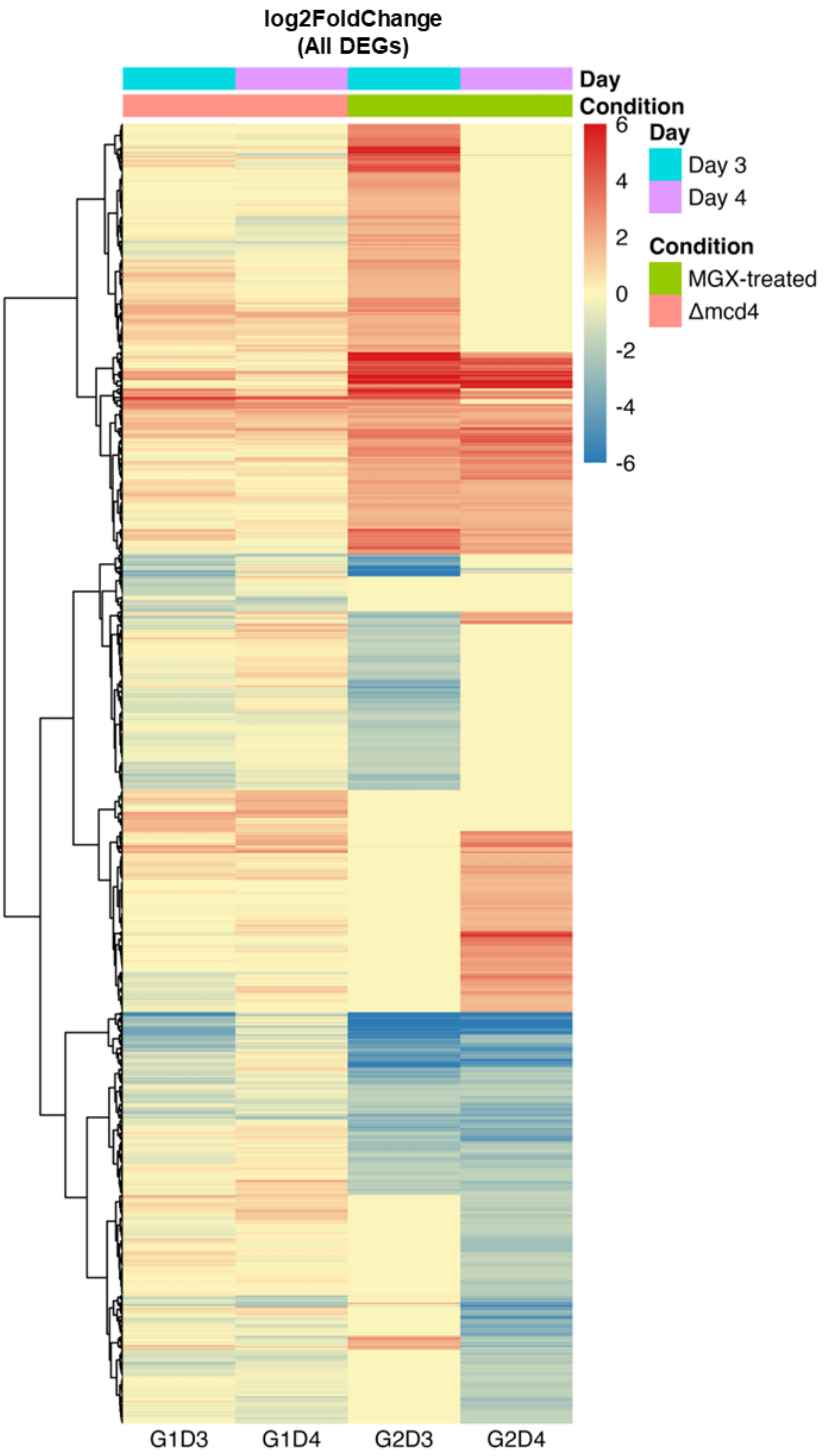

Supplementary Fig. 5: Heatmap of DEGs identified in Δ*mcd4* and MGX-treated AO1 relative to AO1 on days 3 and 4, showing distinct DEGs cluster with shared expression patterns across groups and unique expression patterns under specific conditions.

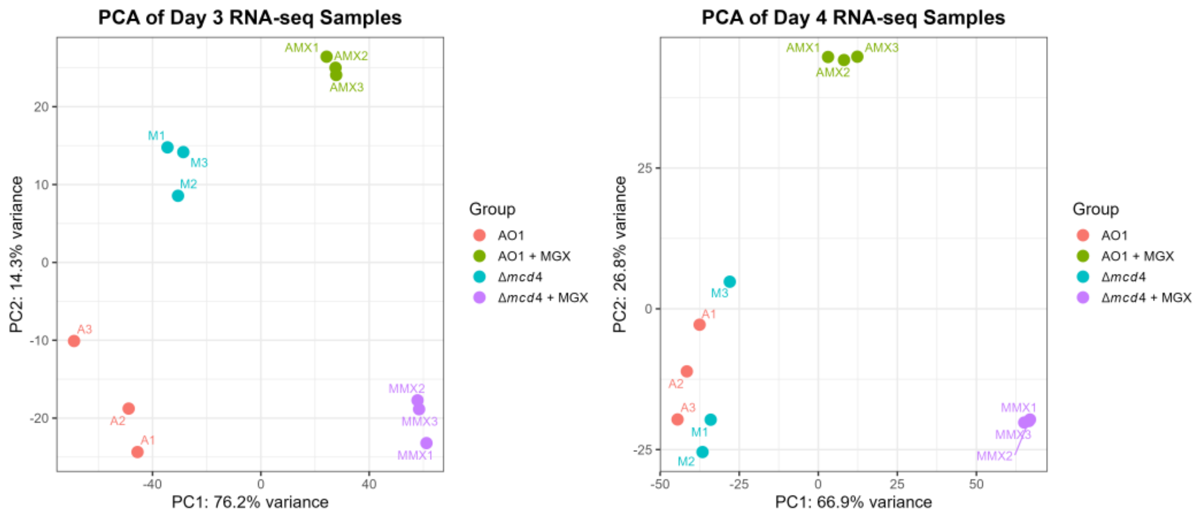

Supplementary Fig. 6: Principal component analysis (PCA) showing the clustering of biological triplicates and distinct separation between groups (AO1, AO1 + MGX, Δ*mcd4* and Δ*mcd4* + MGX), except in AO1 and Δ*mcd4* on day 4.

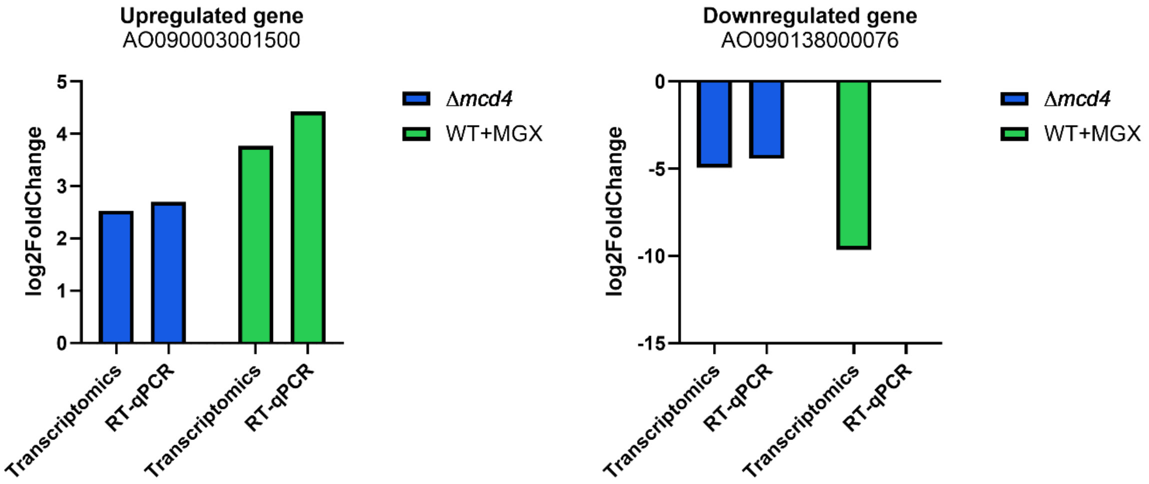

Supplementary Fig. 7: Comparison of log_2 fold change values for an upregulated gene encoding α-1,3-glucan/ α-1,4-glucan synthase (AO090003001500) and a downregulated gene encoding stress response protein (AO090138000076) measured by RT-qPCR and RNA-seq (Novogene). RNA samples were collected from Δ*mcd4* and MGX-treated AO1 cultures on day 3.

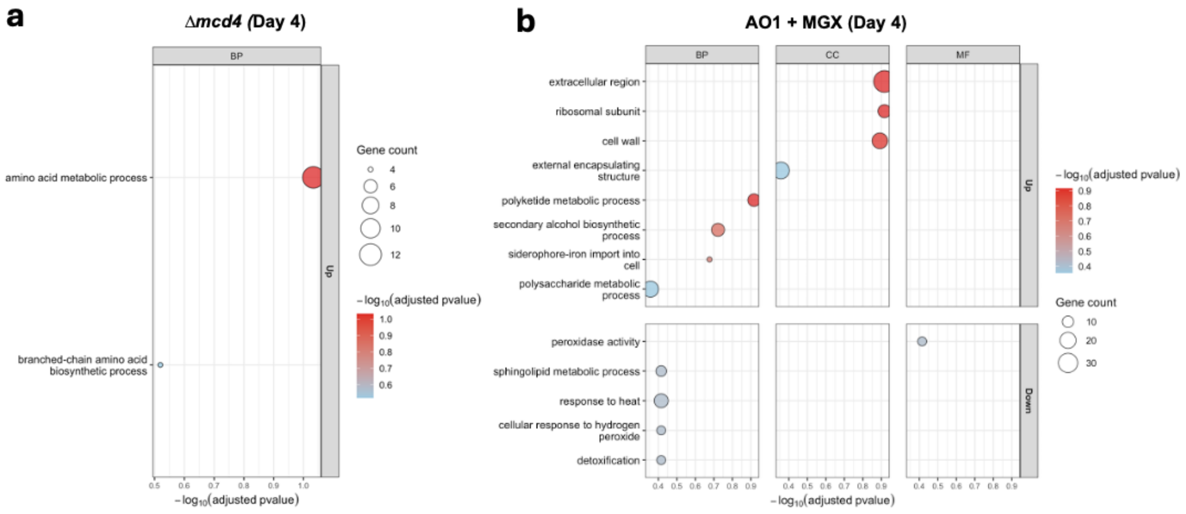

Supplementary Fig. 8: GO enrichment plot from over-representation analysis (ORA) conducted on DEGs identified in (a) Δ*mcd4* and (b) MGX-treated AO1 relative to AO1 on day 4. Enriched GO terms were filtered by REVIGO and GO terms with dispensability < 0.5 are displayed in bubble plot. Bubble size represents the gene number while the colour reflects the p-value.

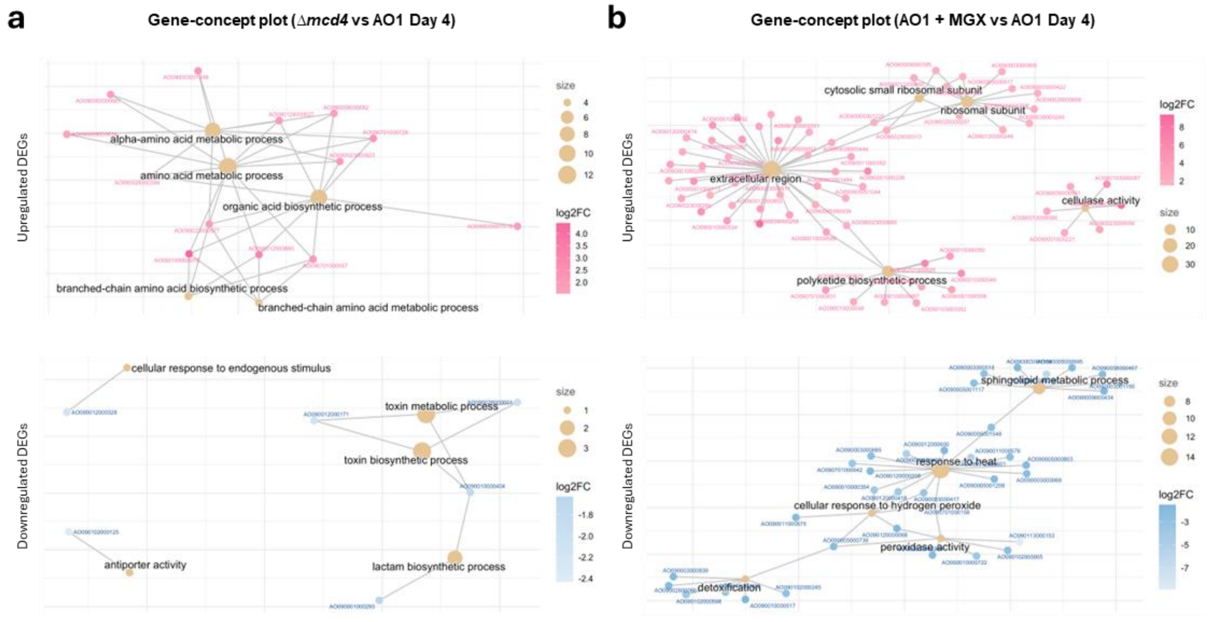

Supplementary Fig. 9: Gene–concept networks highlighting DEGs associated with multiple enriched GO terms in (a) *Δmcd4* and (b) MGX-treated AO1 relative to AO1 on day 4. Lines

connect shared genes among terms. Each beige node represents a GO term, and node size reflects the number of associated genes. The colour of the gene nodes represents the log_2FC.

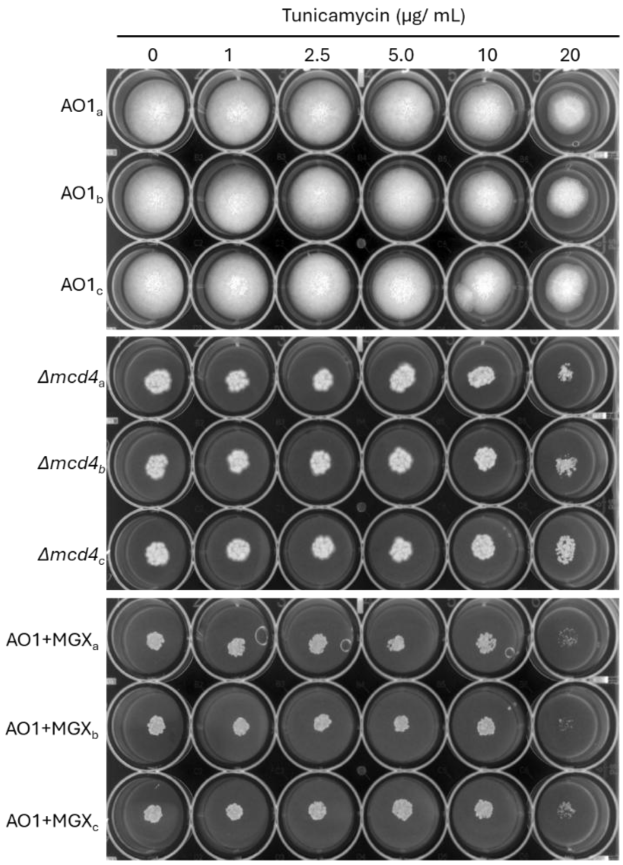

Supplementary Fig. 10: Susceptibility of GPI-AP-perturbed strains to ER stress induced by tunicamycin. Conidia (10^2) were inoculated in the centre of PDA agar treated with 0 − 20 µg/mL of tunicamycin and incubated at 30°C for 2 days.

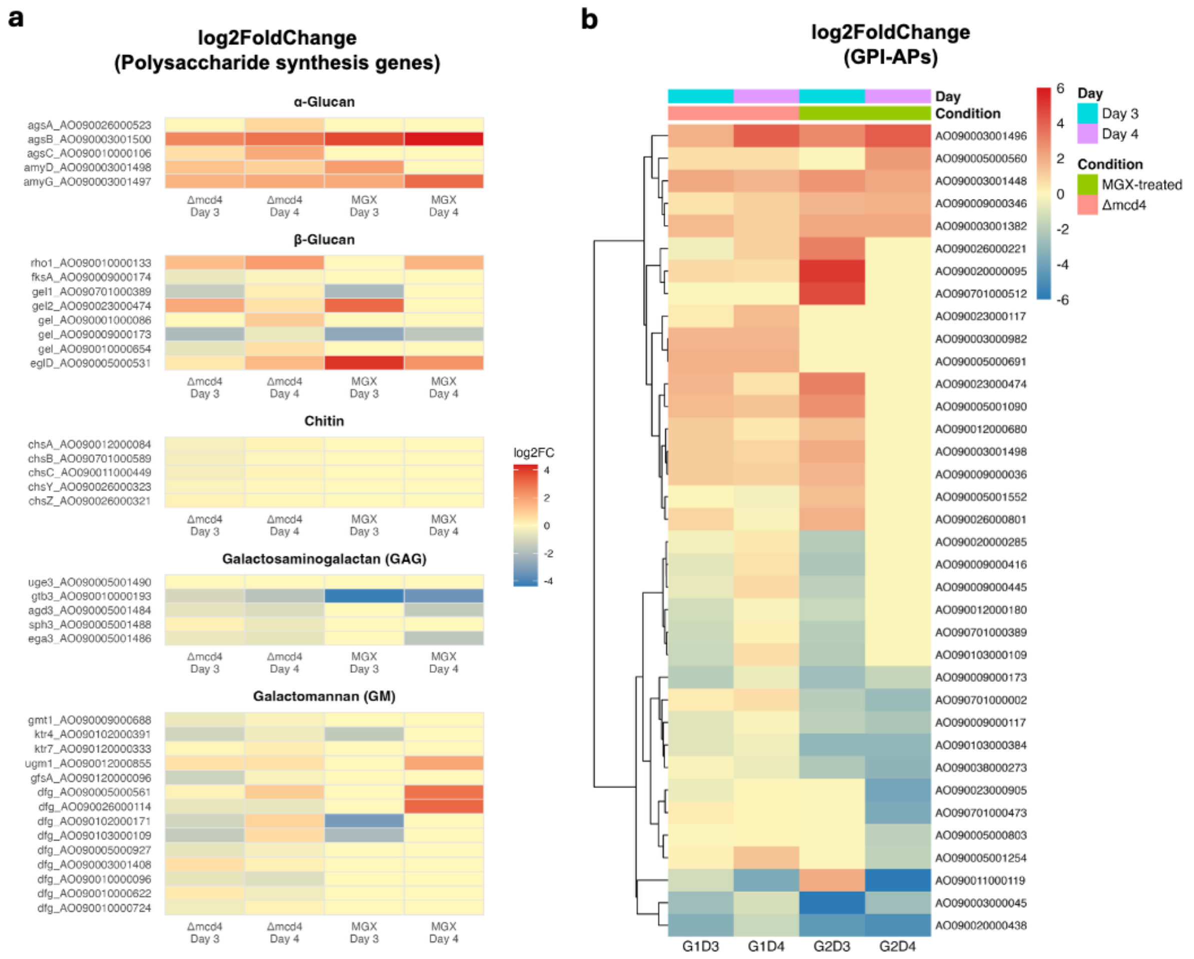

Supplementary Fig. 11: Genetic and chemical perturbation of GPI-anchor biosynthesis results in similar gene regulation in genes responsible for polysaccharides synthesis and genes encoding GPI-anchored proteins. (a) Heatmap of $\log_2$ fold change expression of genes involved in α-glucan, β-glucan, chitin, galactosaminogalactan (GAG) and galactomannan (GM) synthesis. (b) Heatmap of $\log_2$ fold change expression of genes predicted to be GPI-anchored proteins.

Supplementary Table 1: Predicted GPI-anchored proteins in *A. oryzae* from *in silico* screening of the complete proteome on UniProt and FungiDB using SignalP-6.0 and NetGPI-1.1.

Gene ID	UniProt ID	Function
AO090010000654	Q2TW91	1,3-beta-glucanosyltransferase
AO090023000474	Q2UHE9	
AO090001000086	Q2UP74	
AO090701000389	Q2U8L0	
AO090009000173	Q2UUU5	
AO090009000416	Q2UU86	Acid phosphatase PHOa
AO090003001498	Q2UIS5	Alpha-amylase
AO090012000663	Q2UCB4	Cell wall galactomannoprotein
AO090005001552	Q2UPQ5	Cellulose-binding GDSL lipase/acylhydrolase
AO090701001161	A0A1S9DR22	CFEM domain-containing protein
AO090023000216	Q2UI27	
AO090003000045	Q2UMD2	
AO090103000384	Q2TY56	
AO090023000239	Q2UI04	Chitin deacetylase
AO090102000586	Q2UA11	Chitinase
AO090020000438	Q2U490	Copper acquisition factor BIM1-like domain-containing protein
AO090120000174	Q2U6M6	
AO090026000221	Q2UFG2	Cupredoxin
AO090005000560	Q2US58	GH16 domain-containing protein
AO090020000289	Q2U4L7	Glutaminase A
AO090120000279	Q2U6E0	Glycosidase
AO090005001090	Q2UQV1	
AO090001000511	Q2UN44	
AO090005001344	Q2UQ93	
AO090005000538	Q2US76	Glycoside hydrolase family 71
AO090023000915	Q2UGA5	GPI anchored cell wall protein
AO090010000666	Q2TW83	GPI anchored protein
AO090011000916	Q2TZC0	
AO090011000201	Q2U125	
AO090038000286	Q2U2X0	
AO090701000382	Q2U8L4	
AO090012000878	Q2UBT1	
AO090003001448	Q2UIW6	
AO090003001326	Q2UJ78	
AO090003001282	Q2UJB9	
AO090003000674	Q2UKU3	
AO090005000610	Q2US13	
AO090009000445	Q2UU60	
AO090009000346	Q2UUE5	GPI anchored serine-rich protein
AO090020000095	Q2U533	GPI anchored serine-threonine rich protein
AO090012000922	Q2UBP2	
AO090005000803	Q2URJ6	
AO090023000905	Q2UGB5	GPI-anchored cell wall organization protein Ecm33
AO090003000982	Q2UK24	GPI-anchored domain-containing protein
AO090011000119	Q2U193	Hydrophobic surface binding protein A-domain-containing protein
AO090020000588	Q2U3W7	Hydrophobin
AO090701000473	Q2U8D2	Lysophospholipase
AO090012000680	Q2UCA1	
AO090023000685	Q2UGW4	
AO090003001408	Q2UJ03	Mannan endo-1,6-alpha-mannosidase

AO090010000096	Q2TXL6	
AO090103000109	Q2TYU3	
AO090009000148	Q2UUW3	Peptidase A1 domain-containing protein
AO090005001254	Q2UQG8	
AO090012000910	Q2UBQ4	PLC-like phosphodiesterase
AO090701000002	Q8NKB6	Probable aspartic-type endopeptidase opsB
AO090023000083	Q2UIE6	Probable endo-1,3(4)-beta-glucanase
AO090009000117	Q2UUZ1	Probable glucan endo-1,3-beta-glucosidase
AO090010000729	Q2TW27	TSPc domain-containing protein
AO090038000622	Q2U232	Uncharacterized protein
AO090038000273	Q2U2Y1	
AO090020000279	Q2U4M6	
AO090701000512	Q2U8A1	
AO090102000201	Q2UAW9	
AO090012000664	Q2UCB3	
AO090012000359	Q2UD16	
AO090012000180	Q2UDG6	
AO090012000013	Q2UDV5	
AO090026000801	Q2UE13	
AO090023000117	Q2UIC0	
AO090003001496	Q2UIS7	
AO090003001382	Q2UJ25	
AO090005001359	Q2UQ79	
AO090005000691	Q2URU0	
AO090005000652	Q2URX6	
AO090009000467	Q2UU44	
AO090009000036	Q2UV57	
AO090020000285	Q2U4M0	WSC domain-containing protein

Supplementary Table 2: Relative molar composition of the rigid and mobile cell wall polysaccharides in *A. oryzae* AO1 and MGX-treated AO1, Δ*mcd4* and MGX-treated Δ*mcd4*. Molar compositions were determined from the integrated intensities of well-resolved signals corresponding to specific carbon sites in 2D ^{13}C-^{13}C CORD (rigid) and J-mediated INADEQUATE (mobile) components. Values are the average percentages, with errors presenting standard errors derived from spectra analysis. / Represents undetected.

**Rigid**	**AO1**	**AO1 + MGX**	**Δ*mcd4***	**Δ*mcd4* + MGX**
B	34±11	19±4	32±12	24±7
G	2±0.1	3±0.1	2±0	4±0.2
Ch^a	25±6	24±7	16±4	24±7
Ch^b	5±0.2	16±7	13±3	16±4
A^a	21±4	21±5	27±12	18±5
A^b	8±2	14±3	7±1	11±3
Mn	5±0.01	3±0.1	3±0	3±0.1

**Mobile**	**AO1**	**AO1 + MGX**	**Δ*mcd4***	**Δ*mcd4* + MGX**

Gal*f*	25±11	25±8	25±9	15±3
Mn^{12}	11±6	7±1	8±1	8±1
Mn^{16}	13±2	3±0	9±1	9±1
B	4±0.4	14±2	20±0	9±1
B^{Br}	15±5	15±4	18±6	15±3
A	9±1	17±5	7±1	19±5
Gal	11±1	14±3	5±1	11±2
GalN	4±0.2	/	2±0.1	/
GalNAc	8±0	5±0	6±0.5	7±1
Ch	/	/	//	7±0.5

Supplementary Table 3: Transcription levels of cell-cell fusion genes under GPI-AP-perturbed conditions.

**Gene name in *Neurospora crassa***	**Ortholog in *A. oryzae***	**log2FC**			
		**Δ*mcd4* vs AO1 Day 3**	**Δ*mcd4* vs AO1 Day 4**	**AO1 + MGX vs AO1 Day 3**	**AO1 + MGX vs AO1 Day 4**
adv-1	AO090003001259	-3.8724	-1.2224	-5.8027	-4.5661
ada-3	AO090003000967	1.6067	0.7586	n.d.	n.d.
so (ham-1)	AO090003000023	-1.4770	-0.8753	-1.7067	n.d.
ham-5	AO090113000103	-1.7663	-1.3016	-1.6390	-3.8198
ham-9	AO090012000944	-1.5312	-1.3417	-2.3966	-3.5881
nor-1	AO090011000671	-1.2401	-1.2049	-1.8762	-1.9453
nox-1	AO090003000460	-0.6019	-0.1413	n.d.	-1.7526
ham-6	AO090003000459	-0.6149	-0.4799	-1.6129	-2.8647
ham-7	AO090020000438	-3.4307	-1.4840	-4.5398	-4.9958
ham-8	AO090026000826	-2.9644	-1.4804	-4.1338	-4.5088
lfd-2	AO090026000798	-1.2996	-0.7626	n.d.	-1.5358

n.d.: not detected

Supplementary Table 4: Strains and plasmids used in this work

**Strains/ Plasmids**	**Description**	**Source**
A. oryzae		
AO1	RIB40 Δ*wA*::amyB-Lys-Arg-HLY	[27]
Δ*mcd4*	*mcd4* deletion in AO1	This study
Δ*agd3*	*agd3* deletion in AO1	This study
**Plasmids**		
pHT001	*E. coli-Aspergillus* shuttle vector (Takara pPTR II) containing Cas9, ARS1 and TRP1	[27]
pHT001-Δ*mcd4*	pHT001 plasmid containing two sgRNA expression cassettes targeting *mcd4* for CRISPR/Cas9-mediated deletion	This study
pHT001-Δ*agd3*	pHT001 plasmid containing two sgRNA expression cassettes targeting *agd3* for CRISPR/Cas9-mediated deletion	This study

Supplementary Table 5: Primers and sgRNA used in this work

Primer	Sequence	Usage
HT157	CTAAAAC**ACACGCGAGTTCCCACCGAA**ACTTGTTCT TCTTTACAATGATTTATTTACCC	Reverse primer for *mcd4* sgRNA1
HT158	AACAAGT**TTCGGTGGGAACTCGCGTGT**GTTTTAGA GCTAGAAATAGCAAGTTAAAATAAG	Forward primer for *mcd4* sgRNA1
HT159	CTAAAAC**CGTGTGTGTACCTGGGACTG**ACTTGTTCT TCTTTACAATGATTTATTTACCC	Reverse primer for *mcd4* sgRNA2
HT160	AACAAGT**CAGTCCCAGGTACACACACG**GTTTTAGA GCTAGAAATAGCAAGTTAAAATAAG	Forward primer for *mcd4* sgRNA2
HT254	GCAGTGTAACGGCGTATCATCCCTGG	Forward primer to check for *mcd4* deletion
HT255	GGGCTTCCATACCTGCGCACCCATA	Reverse primer to check for *mcd4* deletion
HT468	CTAAAAC**GTGTCAGTACGACAGGCCTC**ACTTGTTCT TCTTTACAATGATTTATTTACCC	Reverse primer for *agd3* sgRNA1
HT469	AACAAGT**GAGGCCTGTCGTACTGACAC**GTTTTAGA GCTAGAAATAGCAAGTTAAAATAAG	Forward primer for *agd3* sgRNA1
HT470	CTAAAAC**TTGGCCAGTCTCACCGTTGA**ACTTGTTCT TCTTTACAATGATTTATTTACCC	Reverse primer for *agd3* sgRNA2
HT471	AACAAGT**TCAACGGTGAGACTGGCCAA**GTTTTAGA GCTAGAAATAGCAAGTTAAAATAAG	Forward primer for *agd3* sgRNA2
HT486	TCCACGATGCTGACTAGGGA	Forward primer to check for *agd3* deletion
HT487	GTGGTCTAGGCCGGTCATTC	Reverse primer to check for *agd3* deletion

Note: sgRNA sites are in **bold**.
